# Associations Between Depression and Reduced Quality of Life in Women with Non-Radiographic Axial Spondyloarthritis: A Cross-Sectional Study

**DOI:** 10.3390/biomedicines14020389

**Published:** 2026-02-08

**Authors:** Marija Rogoznica Pavlović, Mislav Radic, Andrej Belančić, Kristina Skroče, Karla Vurić, Tatjana Kehler

**Affiliations:** 1 Department of Rheumatology, Hospital for Medical Rehabilitation of Heart and Lung Diseases and Rheumatism ‘Thalassotherapia Opatija’, 51410 Opatija, Croatia; karla.vuric@gmail.com (K.V.);; 2 Department of Internal Medicine, Division of Rheumatology, Allergology and Clinical Immunology, Center of Excellence for Systemic Sclerosis in Croatia, University Hospital of Split, 21000 Split, Croatia; mislavradic@gmail.com; 3 Internal Medicine Department, School of Medicine, University of Split, 21000 Split, Croatia; 4 Department of Basic and Clinical Pharmacology and Toxicology, Faculty of Medicine, University of Rijeka, 51000 Rijeka, Croatia; a.belancic93@gmail.com; 5 Faculty of Medicine, University of Rijeka, 51000 Rijeka, Croatia; 6 Faculty of Health Studies, University of Rijeka, 51000 Rijeka, Croatia

**Keywords:** anxiety, depression, female, non-radiographic axial spondyloarthritis, quality of life

## Abstract

**Background/Objectives**: Axial spondyloarthritis (axSpA) is a chronic systemic inflammatory disease that adversely affects both physical and mental health. This cross-sectional study aimed to examine the associations between spondyloarthritis features (SpA-fs) and disease-related variables (DRVs: disease duration, Visual Analogue Scale, Ankylosing Spondylitis Disease Activity Score [ASDAS], Bath Ankylosing Spondylitis Disease/Functional Activity Index), as well as potential correlations with quality of life (QoL) and symptoms of anxiety and depression in women with non-radiographic axSpA (nr-axSpA). **Methods**: This study included 78 women with nr-axSpA. Data were obtained from medical records and assessed using two validated instruments: the Short Form-36 (SF-36) and the Hospital Anxiety and Depression Scale (HADS). **Results**: The mean age of the cohort was 39.8 ± 7.8 years, with a mean disease duration of 4.80 ± 5.37 years and a mean ASDAS of 2.09 ± 1.14. DRVs, correlated positively with HADS scores and negatively with SF-36 scores. Patients with family histories of SpA had significantly lower mental-component SF-36 scores and higher HADS-D scores. Lower quality of life was associated with DRVs, particularly disease duration. Significant associations with depressive symptoms were observed for both SpA features and DRVs. **Conclusions**: In women with nr-axSpA, both SpA-fs and DRVs are associated with reduced QoL and elevate the risk of anxiety and depression, underscoring the need for thorough patient evaluation that encompasses psychological health.

## 1. Introduction

Axial spondyloarthritis (axSpA) is a chronic rheumatic disease marked by inflammation of the sacroiliac joint, spine and entheses [1]. The Assessment of Spondyloarthritis International Society (ASAS) classifies axSpA into two subtypes: the radiographic form, previously known as Ankylosing Spondylitis (AS), and the non-radiographic form (nr-axSpA) [2,3]. Nr-axSpA is considered an initial stage of the disease, characterized by the absence of definitive radiographic sacroiliitis [3]. The onset of axSpA usually happens in early adulthood, which is a big personal and social problem [1]. The ratio of men to women is different in each group. In AS, there are two men for every woman, but in nr-axSpA, there is only one man for every woman [4]. This may suggest that women experience structural changes more slowly or less frequently than men for reasons that remain inadequately understood [4].

Patients with axSpA may exhibit various spondyloarthritis features (SpA-fs), including inflammatory back pain (IBP), arthritis, enthesitis (heel), uveitis, dactylitis, psoriasis, inflammatory bowel disease (IBD), elevated C-reactive protein (CRP), Human Leukocyte Antigen B27 (HLA-B27) positivity, and a favorable response to non-steroidal anti-inflammatory drugs (NSAIDs) [2,3].

Along with musculoskeletal problems, patients frequently experience fatigue, sleep disturbances, depression, anxiety, and stress, all of which detrimentally impact daily functioning and quality of life (QoL) [5,6,7,8,9]. Over the past twenty years, there has been a lot of interest in finding ways to improve patients’ QoL, especially for those with long-term illnesses [10]. QoL is an important measure for improving health and happiness. A chronic, progressive condition such as axSpA may lead to numerous psychological symptoms, predominantly anxiety and depression [11]. Reported prevalence rates vary between 15% and 52% for depression and 23% and 40% for anxiety [12,13].

Researchers have previously examined the correlation between QoL and disease activity or functional status in patients with AS [14,15,16,17], and they have also investigated the impact of SpA on symptoms of anxiety and depression [12,13,18,19]. Numerous studies have explored individual SpA-fs, including enthesitis, psoriasis, and IBD, and their effects on both QoL and the prevalence of anxiety and depression [15,20,21,22,23]. However, the aggregate influence of multiple SpA-fs has received limited attention, and even fewer studies have evaluated their collective impact on specific QoL domains and symptoms of anxiety and depression in women with nr-axSpA [24,25].

The aim of the present study was to determine the influence of multiple SpA-fs, radiographic changes and disease-related variables (DRVs) on the QoL and symptoms of anxiety and depression in women with nr-axSpA.

## 2. Materials and Methods

### 2.1. Study Objective

The objective of this study was to investigate factors (SpA-fs, radiographic changes and DRVs) associated with impaired QoL domains and symptoms of anxiety and depression in female patients with nr-axSpA.

### 2.2. Study Design

A cross-sectional study design was employed. Ethical approval was obtained from the Ethics Committee of Thalassotherapia Opatija Hospital, Opatija, Croatia (01-000-00-329/2). Written informed consent was obtained from all participants prior to enrollment. This study was conducted in accordance with the principles of the Declaration of Helsinki.

### 2.3. Participants

A total of 78 female, sexually active patients with nr-axSpA, of >18 years of age, were included in this study. The diagnosis of nr-axSpA was established according to the ASAS classification criteria [2,3,26]. Recruitment was carried out at the Department of Rheumatology, Thalassotherapia Opatija Hospital (Opatija, Croatia), between January 2022 and October 2023.

### 2.4. Exclusion Criteria

Exclusion criteria comprised the following: AS; other rheumatic diseases; fibromyalgia; severe hypertension; diabetes mellitus; neurological, malignant, or hematological disorders; endometriosis; congenital genetic disorders; known psychiatric illness; and substance abuse. Patients receiving antihypertensive, antidepressant, anxiolytic, antipsychotic, or antiepileptic medications were also excluded due to their common adverse effects [27].

### 2.5. Clinical Assessment

Diagnosis of nr-axSpA required either:

1. Sacroiliitis on imaging, defined as active inflammation on magnetic resonance imaging (MRI) highly suggestive of sacroiliitis associated with plus ≥ 1 SpA-fs, or
2. HLA-B27 positivity plus ≥ 2 SpA-fs.

#### 2.5.1. Collected Variables and Outcome Measures

Data regarding demographics, medical history, treatment (NSAIDs, Disease-Modifying Antirheumatic Drugs [DMARDs], biologics), and radiographic findings (sacroiliitis and/or spondylitis on MRI) were collected. Information was obtained from electronic medical records and structured questionnaires.

Disease activity was assessed using the Bath Ankylosing Spondylitis Disease Activity Index (BASDAI) and the Ankylosing Spondylitis Disease Activity Score (ASDAS). An ASDAS ≥ 2.1 or a BASDAI ≥ 4 meant that the disease was active [28,29]. The Bath Ankylosing Spondylitis Functional Index (BASFI) was used to measure functional status [30]. A 100 mm Visual Analogue Scale (VAS) was used to measure the intensity of pain.

The erythrocyte sedimentation rate (ESR), which was measured by the Westergren method (mm/h), and CRP levels, which were measured by nephelometry (mg/dL), were both signs of inflammation. The presence of HLA-B27 was noted.

The disease-related variables examined included VAS pain, BASDAI, ASDAS, BASFI, and disease duration. The presence of SpA-fs was determined by a rheumatologist and based on clinical history.

#### 2.5.2. Questionnaires

This study utilized two validated patient-reported outcome measures: the Short Form-36 Health Survey (SF-36) and the Hospital Anxiety and Depression Scale (HADS) [31,32]. All questionnaires were administered in their Croatian versions, which have been validated in previous research [31,32,33,34]. All participants successfully completed the questionnaires.

##### Short Form-36 Health Survey

The Medical Outcomes Study SF-36 is a widely used generic tool for measuring health-related QoL [35]. The questionnaire comprises 36 items across eight domains: physical functioning, physical role, bodily pain, general health, vitality, social functioning, emotional role, and mental health. The first four domains reflect physical health, whereas the remaining four reflect mental health. Scores for each domain are converted to a 0 to 100 scale, with higher scores indicating better health. Two summary scores are derived: the Physical Component Summary (PCS) and the Mental Component Summary (MCS). All SF-36 domain results were transformed into norm-based T-scores based on Croatian normative data (mean = 50, SD = 10). The use of national reference values enabled comparisons of the study sample to the general Croatian population [33]. The SF-36 has undergone extensive international validation, including in patients with rheumatic diseases, and the Croatian version was translated and validated as part of the International Quality of Life Assessment Project [33,36,37,38].

##### Hospital Anxiety and Depression Scale (HADS)

The HADS is a self-report tool that measures anxiety (HADS-A) and depression (HADS-D) symptoms from the previous week [39]. There are seven items in each subscale, and the scores can be anywhere from 0 to 21. Scores between 8 and 10 suggest mild symptoms, while scores of 11 or higher suggest clinically significant anxiety or depression [40]. A cut-off value of ≥8 has been shown in many studies to be the best balance of sensitivity and specificity [41,42,43]. The HADS is a commonly utilized screening tool in both primary care and hospital environments, having been validated across various populations [40,41,44].

### 2.6. Statistical Analysis

Statistical analyses were performed using JASP v0.18.3 for Mac (University of Amsterdam, The Netherlands), Microsoft Excel 2024 for Mac (version 16.91; Microsoft Corporation, Redmond, WA, USA), and MedCalc v12.1.3 (MedCalc Software bvba, Ostend, Belgium).

Categorical variables were summarized as absolute and relative frequencies (n, %). Continuous variables were presented as means ± standard deviation (SD) or medians with interquartile ranges (IQRs; 25th–75th percentiles), depending on data distribution. Normality was evaluated using skewness, kurtosis, and the Shapiro–Wilk test.

Associations between variables were examined using Pearson’s or Spearman’s correlation coefficient, according to the distributional properties of the data. Differences in outcomes between groups defined by associated factors were evaluated using either an unpaired *t*-test or the Mann–Whitney test, as appropriate. Equality of variance was assessed with an F-test.

Multivariable linear regression analyses were performed to identify patient characteristics independently associated with reduced QoL and symptoms of anxiety and depression.

A two-tailed *p*-value < 0.05 was considered statistically significant.

## 3. Results

### 3.1. Demographic Characteristics of Participants

Of the 85 female patients with nr-axSpA who were invited to participate, 78 (response rate: 92%) completed the questionnaires and were included in the present analyses. The mean age of the participants was 39.8 ± 7.8 years.

### 3.2. Disease-Related Variables of, Treatment of, and Radiographic Changes in nr-axSpA

The mean disease duration was 4.80 ± 5.37 years. On average, disease activity was moderate, as indicated by the ASDAS (2.09 ± 1.14) and BASDAI (3.16 ± 1.84). Functional status, assessed by the BASFI, averaged 1.72 ± 1.56, and pain intensity, measured by the VAS, was 42.6 ± 13.5 mm, with considerable variability among participants.

Laboratory findings showed a mean ESR of 11.63 mm/h and CRP of 4.12 mg/dL. Regarding treatment, nearly all patients were receiving NSAIDs, more than half were treated with biologics and approximately one-third received DMARDs. MRI abnormalities were present in more than half of the cohort, with sacroiliitis observed in 49% and spondylitis in 17% of patients. A complete overview of clinical characteristics is presented in Table 1.

### 3.3. Spondyloartritis Features of nr-axSpA

All patients presented with IBP and demonstrated a good response to NSAIDs. More than half of the cohort had peripheral arthritis, while approximately one-third exhibited enthesitis, a family history of SpA, HLA-B27 positivity, or elevated CRP levels. Dactylitis was present in three patients, psoriasis in four, and inflammatory bowel disease (IBD) in two patients. None of the participants had uveitis. A detailed overview of SpA-fs in the study cohort is presented in Table 2.

### 3.4. Changes in QoL and Symptoms of Anxiety and Depression in nr-axSpA

QoL outcomes were evaluated using the SF-36 and yielded a median PCS score of 43.04 (IQR = 39.4–47.34), indicating a moderate reduction in physical health relative to population norms (T = 50). The MCS score median, 46.16 (IQR = 40.26–54.28), reflects a slightly below-average level of mental health with greater inter-individual variability.

The HADS-A score showed a normal distribution (*p* > 0.051), while the HADS-D score was non-normally distributed (*p* < 0.01). The HADS-A score mean, 8.52 ± 3.62, and HADS-D score median, 6 (IQR = 4–9), respectively, suggest mild symptoms in the study population.

### 3.5. Correlations Between SF-36, HADS and Disease-Related Variables in nr-axSpA

In the study cohort, Pearson’s and Spearman’s correlation coefficients were applied according to data distribution. The PCS showed negative correlations with the VAS, BASDAI and ASDAS. Disease duration showed a borderline association with lower PCS scores (*p* = 0.05). Negative correlations were also observed with the MCS and the BASDAI, ASDAS and BASFI.

Positive correlations were observed between the HADS-A and the BASDAI and ASDAS, as well as the HADS-D and the ASDAS, BASDAI, and BASFI. A complete overview of the correlation analyses is presented in Table 3.

### 3.6. Differences in Outcomes Between Predictor-Based Groups

Factors included in the analyses were arthritis, enthesitis, family history of SpA, HLA-B27 and elevated CRP. Uveitis, psoriasis, dactylitis, and IBD were excluded due to small sample sizes. Outcomes were PCS, MCS and HADS scores. Depending on the distribution of the variables, an unpaired t-test and the Mann–Whitney U test were used for group comparisons.

Statistically significant differences in the MCS and HADS-D, along with a borderline difference in the HADS-A, were observed among participants with positive family histories of SpA. Furthermore, participants with elevated CRP demonstrated a borderline difference in the MCS compared with those with normal CRP levels. All results of group comparisons are presented in Table 4 and Table 5.

### 3.7. Factors Associated with Differences in QoL and Symptoms of Anxiety and Depression in Female Patients with nr-axSpA

Multiple linear regression models were performed to evaluate SpA-fs as factors associated with the PCS and MCS. The models were not statistically significant (R^2^ = 0.09; F (5,72) = 1.11; *p* = 0.366) for either the PCS or the MCS (R^2^ = 0.16; F (5,72) = 1.96; *p* = 0.099). None of the included associated factors showed significant association with the PCS and MCS.

Separate regression models were used to evaluate radiographic changes as associations with the PCS and MCS. These models were also not statistically significant (R^2^ = 0.03; F (2,75) = 0.04; *p* = 0.398) for either the PCS or the MCS (R^2^ = 0.05; F (2,75) = 1.50; *p* = 0.232), indicating that radiographic changes did not explain a meaningful proportion of variance in the PCS and MCS.

In contrast, the models evaluating DRVs associated with the PCS and MCS were statistically significant (PCS—R^2^ = 0.19; F (5,72) = 2.44; *p* = 0.046 and MCS—R^2^ = 0.19; F (5,72) = 2.63; *p* = 0.043), indicating that DRVs explained approximately 19% of the variance in both outcomes. Within the PCS model, disease duration was identified as a significant associated factor (β = 0.02, 95% CI [0.00, 0.03], *p* <0.05), while none of the other DRVs showed significant associations with either the PCS or MCS.

All regression results are presented in Table 6.

The regression model evaluating SpA-fs associated with the HADS-A was not statistically significant (R^2^ = 0.15; F (5,72) = 1.86; *p* = 0.117). In contrast, the model for the HADS-D was statistically significant (R^2^ = 0.21; F (5,72) = 2.88; *p* = 0.023), indicating that SpA-fs explained approximately 21% of the variance in the HADS-D. None of the included associated factors showed significant independent association with either the HADS-A or HADS-D.

Separate regression models were used to evaluate radiographic changes as factors associated with HADS scores. These models were not statistically significant for either the HADS-A (R^2^ = 0.01; F (2,75) = 0.23; *p* = 0.797) or HADS-D (R^2^ = 0.03; F (2,75) = 0.91; *p* = 0.41), indicating that radiographic changes did not explain a meaningful proportion of variance in HADS scores.

Multiple linear regression models were performed to evaluate DRVs as factors associated with HADS scores. The model of HADS-A scores was not statistically significant (R^2^ = 0.16; F (5,72) = 1.96; *p* = 0.1). In contrast, the model for the HADS-D was statistically significant (R^2^ = 0.31; F (5,72) = 4.66; *p* = 0.001), explaining approximately 31% of the variance in the HADS-D. None of the included factors showed a significant independent association with either the HADS-A or HADS-D.

All regression results are presented in Table 7.

## 4. Discussion

### 4.1. Quality of Life

AxSpA is a long-term disease that makes women’s QoL worse. In our study, DRVs (VAS, BASDAI, ASDAS, BASFI) exhibited a negative correlation with both PCS and MCS scores, aligning with the hypothesis that increased disease activity correlates with diminished QoL. A borderline association was noted between disease duration and the PCS. In exploratory multivariable analyses, DRVs collectively exhibited a significant association with SF-36 scores. Among the individual variables, an extended disease duration was correlated with a diminished physical QoL. Nonetheless, due to the cross-sectional design, restricted sample size, and marginal results in univariate analyses, this observation must be regarded with caution.

These results demonstrate that elevated disease activity scores correlate with diminished QoL scores, consistent with previous research [1,45]. Yang et al. [1] showed that the BASDAI and BASFI were very negatively correlated with some parts of the SF-36. They concluded that people with AS had much lower QoL than the general population. Cui et al. [45] similarly discovered that elevated disease activity correlated with diminished SF-36 scores, alongside heightened anxiety, depression, and sleep disturbances in patients with axSpA.

In our study, patients with family histories of SpA exhibited markedly lower MCS scores in comparison to those lacking such characteristics. This finding indicates that having affected family members may adversely impact psychological well-being, potentially due to increased awareness of the long-term disease trajectory and possible complications, which may complicate coping mechanisms. This interpretation aligns with the existing literature that suggests chronic illnesses can diminish quality of life for both patients and their family members, as evidenced by the research conducted by Snyder et al. [46].

### 4.2. Anxiety, Depression, and Psychological Burden

Patients with axSpA frequently experience mental health issues, such as anxiety and depression [12,13]. Prior studies have shown that heightened disease severity and diminished QoL correlate with elevated psychological distress [18]. Consequently, the findings of the present study must be interpreted in relation to untreated or mildly symptomatic manifestations of anxiety and depression.

An increased overall disease burden was associated with higher HADS scores, while individual disease activity indices (ASDAS, BASDAI, BASFI) demonstrated positive correlations with both anxiety and depressive symptoms. Since higher HADS scores indicate more severe psychological impairment, these findings suggest that a worse disease status correlates with inferior mental health outcomes.

The multivariable linear regression analysis revealed no independent factors associated with anxiety. Nevertheless, depressive symptoms exhibited a significant association with both the SpA-fs group and the DRV group. Notably, SpA-fs elucidated approximately 21% of the variance in the HADS-D, whereas disease variables represented around 31%. This suggests that depression in females with nr-axSpA may result from a combination of phenotypic SpA traits and increased disease activity.

In a study by Reddy et al., no independent predictors of anxiety were identified, unlike depression, which was present in both sexes [47]. Similarly, Wright et al. [48] found that women with axSpA tend to experience widespread pain and more severe psychological symptoms, particularly depression, than men with axSpA. Zou et al. [19] demonstrated that disease activity scores are associated with the burden of anxiety and depression in both AS and nr-axSpA patients.

However, we found limited evidence in the existing literature indicating that multiple SpA-fs influence the risk of depression especially in patients with nr-axSpA. In our study, patients with histories of SpA had significantly higher HADS-D scores compared with those without these features, suggesting that having affected family members may have a detrimental impact on psychological well-being.

Central sensitization may partially contribute to depressive and anxiety symptoms in this population and warrants further investigation in future studies.

### 4.3. Role of SpA Features

Although the correlations between disease activity and diminished QoL, anxiety, and depression have been extensively documented, our study enhances understanding by assessing the cumulative effect of various SpA-fs on QoL and symptoms of anxiety and depression in women with nr-axSpA, a population that has not been thoroughly examined. Prior studies evaluated the relationship between some SpA-fs, like psoriasis, psoriatic arthritis, IBD and QoL, as well as symptoms of anxiety and depression, but not all of them together in axSpA patients [15,20,21,22,23,49]. Our results found the associations between family history of SpA and MCS and HADS-D scores, emphasizing the multifactorial nature of psychological symptoms in women with nr-axSpA. The SpA-fs group demonstrated significant association values for depression in our participants. Notably, other SpA characteristics (arthritis, enthesitis and HLA-B27 positivity) did not exhibit statistically significant association with QoL domains or psychological outcomes in our study, as they have shown in other studies [50,51,52].

### 4.4. Radiographic Changes

Prior research utilizing QoL and the functional indexes showed them to be correlated with the Bath Ankylosing Spondylitis Radiology Index (BASRI) [53,54,55]. Yacoub et al. [55] revealed the correlation between the domain scores of the SF-36 and the BASRI. Our study revealed no association between radiographic changes (sacroiliitis and spondylitis on MRI) and HADS or QoL scores. This suggests that structural damage alone may not directly influence psychological or QoL outcomes. To our knowledge, no previous studies have investigated MRI-detected changes in axSpA and their effects on these outcomes.

### 4.5. Future Directions

Future studies should try to confirm these results in larger multicenter groups to confirm the links that were found between SpA and clinical variables, QoL, anxiety and depression. Longitudinal studies are needed to figure out the time relationship and possible cause-and-effect relationship between disease activity, structural changes, and psychological outcomes. Also, looking into the effects of psychosocial factors, coping strategies, and family support could help us understand how mental health works in axSpA.

### 4.6. Strengths and Clinical Implications

The strengths of this research include the use of multiple SpA-fs as associated factors of reduced QoL and psychological symptoms in women with nr-axSpA, an area that has been rarely explored in the literature. Studies focusing specifically on women with axSpA are limited, and those investigating women with nr-axSpA are particularly scarce, which underscores the contribution of the present findings. We also used validated assessment tools (SF-36, HADS) and incorporated MRI-confirmed radiographic changes as associated factors. Furthermore, by applying a more stringent HADS cut-off (>11), we enhanced the reliability of identifying clinically significant anxiety and depression.

### 4.7. Limitations

This study has several limitations that should be considered when interpreting the findings. First, its cross-sectional design prevents determinations of causality or temporal associations between disease characteristics and outcomes, including psychological burden and QoL. Longitudinal studies are necessary to elucidate these associations more effectively. Second, the sample size was small and homogeneous, which reduces statistical power in multivariable analyses and increases the risk of type II error; therefore, all regression results should be considered exploratory and indicative of associations rather than conclusive causal relationships. Third, uncommon but clinically significant manifestations of SpA, including uveitis, psoriasis, dactylitis, and IBD, were inadequately represented, limiting the assessment of their impact on patient-reported outcomes. Fourth, recruiting patients from a hospital setting may have introduced selection bias, as individuals with more active or severe disease are more likely to be referred, potentially resulting in an overestimation of the burden of dysfunction and psychological comorbidities compared to community samples. Fifth, participants were exclusively recruited from a single center. Sixth, although patients with formal diagnoses of fibromyalgia were excluded, subclinical central sensitization or widespread pain cannot be disregarded. The absence of targeted instruments for assessing fibromyalgia, generalized pain, or central sensitization represents a considerable limitation, particularly in light of the predominance of female patients and the acknowledged psychological symptom burden. Seventh, patients receiving antidepressant or anxiolytic treatment were excluded, which may have resulted in the underrepresentation of individuals with more severe depression or anxiety. Consequently, the prevalence and severity of depressive symptoms identified in this study may be underestimated, and the findings are predominantly relevant to untreated or mildly affected patients with nr-axSpA.

Finally, the lack of a healthy control group restricts the contextualization of the findings, as it is impossible to ascertain the degree to which the observed impairments surpass those anticipated in the general population.

## 5. Conclusions

Our findings indicate a significant prevalence of diminished QoL, accompanied by mild symptoms of anxiety and depression, in women with nr-axSpA. The overall disease burden and SpA-fs, particularly a family history of SpA, exert a more significant impact on mental health and QoL. These findings underscore the significance of thorough evaluation, prompt recognition, and focused interventions aimed at enhancing psychological well-being and QoL in women with nr-axSpA. Increasing awareness among patients and healthcare professionals is an essential initial step toward enhancing mental health outcomes and overall QoL in this demographic.

## Figures and Tables

**Table 1:** Disease-related variables of, treatment of and radiographic changes in nr-axSpA.

Variables	nr-axSpA N = 78
Clinical characteristics	
Disease duration, years (mean ± SD)	4.80 ± 5.37
IBP, n (%)	78 (100)
ESR (average, mmh^−1^)	11.63
CRP (average, mgdL^−1^)	4.12
VAS, mm (mean ± SD)	42.6 ± 13.5
ASDAS (mean ± SD)	2.09 ± 1.14
BASDAI (mean ± SD)	3.16 ± 1.84
BASFI (mean ± SD)	1.72 ± 1.56
NSAIDs, n (%)	65 (83)
Therapy (* Percentages for medication subgroups were calculated relative to the subjects who exhibited that characteristic.)	
Biological therapy—total, n (%)	45 (58)
TNFi, n (%)	20 (44 *)
IL-17i, n (%)	17 (38 *)
JAKi, n (%)	8 (18 *)
DMARDs, total, n (%)	26 (33.3)
Sulfasalazine, n (%)	19 (73 *)
Methotrexate, n (%)	7 (27 *)
Radiographic changes (* Percentages for radiographic changes were calculated relative to the subjects who exhibited that characteristic.)	
Sacroiliitis—total, n (%)	38 (49)
Bilateral, n (%)	25 (66 *)
Unilateral, n (%)	13 (34 *)
Spondylitis—total, n (%)	17 (17)
Anterior, n (%)	12 (71 *)
Posterior, n (%)	5 (29 *)

Abbreviations: ASDAS—Ankylosing Spondylitis Disease Activity Score; BASDAI—Bath Ankylosing Spondylitis Disease Activity Index; BASFI—Bath Ankylosing Spondylitis Functional Activity Index; CRP—C-reactive protein; DMARDs—Disease-Modifying Antirheumatic Drugs; ESR—erythrocyte sedimentation rate; IBP—inflammatory back pain; IL-17i—Interleukin 17 inhibitor; JAKi—Janus Kinase Inhibitor; nr-axSpA—non-radiographic axial spondyloarthritis; NSAIDs—non-steroidal anti-inflammatory drugs; N—total number; n—number of cases; SD—standard deviation; TNFi—Tumor Necrosis Factor Inhibitor; VAS—Visual Analogue Scale.

**Table 2:** Spondyloarthritis features of nr-axSpA.

Variables	nr-axSpA N = 78
IBP, n (%)	78 (100)
Arthritis, n (%)	43 (55)
Enthesitis (heel), n (%)	28 (36)
Uveitis, n (%)	0 (0)
Dactylitis, n (%)	3 (4)
Psoriasis, n (%)	4 (5)
IBD, n (%)	2 (3)
Good response to NSAIDs, n (%)	78 (100)
Family history of SpA, n (%)	34 (44)
HLA-B27 positivity, n (%)	28 (36)
Elevated CRP (mgdL^−1^), n (%)	22 (28)

Abbreviations: CRP—C-reactive protein; HLA—Human Leukocyte Antigen; IBD—inflammatory bowel disease; IBP—inflammatory back pain; nr-axSpA—non-radiographic axial spondyloarthritis; NSAIDs—non-steroidal anti-inflammatory drugs; N—total number, n—number of cases; SpA—spondyloarthritis.

**Table 3:** Correlation analysis of SF-36, HADS scores and disease-related variables in nr-axSpA.

		VAS	BASDAI	ASDAS	BASFI	Disease Duration
PCS	Spearman’s r	−0.255	−0.361	−0.376	−0.138	0.255
	*p*	0.049	0.005	0.003	0.293	0.05
MCS	Spearman’s r	−0.178	−0.429	−0.398	−0.341	−0.020
	*p*	0.172	<0.001	0.002	0.008	0.878
HADS-A	Pearson’s r	−0.017	0.256	0.317	0.220	0.036
	*p*	0.899	0.048	0.014	0.091	0.782
HADS-D	Spearman’s r	0.139	0.503	0.442	0.450	0.042
	*p*	0.289	<0.001	<0.001	<0.001	0.752

Abbreviations: ASDAS—Ankylosing Spondylitis Disease Activity Score; BASDAI—Bath Ankylosing Spondylitis Disease Activity Index; BASFI—Bath Ankylosing Spondylitis Functional Activity Index; HADS-A—Hospital Anxiety and Depression Scale—Anxiety; HADS-D—Hospital Anxiety and Depression Scale—Depression; MCS—Mental Component Summary; PCS—Physical Component Summary; SF-36—Short Form-36; VAS—Visual Analogue Scale.

**Table 4:** Group differences in PCS and MCS according to SpA-fs.

Associated Factors	Outcomes	Test	Statistics-U	*p*	95% CI	Meaning
Arthritis	PCS	Mann–Whitney	435.5	0.979	−3.12 to 2.86	ns
Enthesitis			309.5	0.207	−0.89 to 5.23	ns
Family History of SpA			357	0.356	−4.29 to 1.71	ns
HLA-B27			269	0.169	−5.83 to 0.88	ns
Elevated CRP			332	0.933	−3.45 to 3.25	ns
Arthritis	MCS	Mann–Whitney	431	0.929	−5.18 to 4.46	ns
Enthesitis			317	0.332	−2.70 to 6.92	ns
Family History of SpA			266	0.019	−9.77 to −0.85	*p* < 0.05
HLA-B27			284	0.262	−8.06 to 2.34	ns
Elevated CRP			223	0.05	0.05 to 10.23	Borderline

Abbreviations: CI—confidence interval; CRP—C-reactive protein; HLA—Human Leukocyte Antigen; MCS—Mental Component Summary; ns—non-significant; PCS—Physical Component Summary; SpA—spondyloarthritis.

**Table 5:** Group differences in HADS scores according to SpA-fs.

Associated Factors	Outcomes	Test	Statistics-U, t	*p*	95% CI	Meaning
Arthritis	HADS-A	Unpaired t-test	1.54	0.128	−0.43 to 0.94	ns
Enthesitis			1.49	0.143	−3.47 to 0.51	ns
Family History of SpA			2.0	0.05	0.00 to 3.79	Borderline
HLA-B27			1.15	0.253	−0.89 to 3.33	ns
Elevated CRP			0.03	0.976	−2.17 to 2.24	ns
Arthritis	HADS-D	Mann–Whitney	401	0.587	−2.00 to 1.00	ns
Enthesitis			312.5	0.222	−3.00 to 1.00	ns
Family History of SpA			244	0.006	1.00 to 5.00	*p* < 0.05
HLA-B27			264.5	0.144	0.00 to 3.00	ns
Elevated CRP			282.5	0.351	−3.00 to 1.00	ns

Abbreviations: CI—confidence interval; CRP—C-reactive protein; HADS-A—Hospital Anxiety and Depression Scale—Anxiety; HADS-D—Hospital Anxiety and Depression Scale—Depression; HLA—Human Leukocyte Antigen; ns—non-significant; SpA—spondyloarthritis.

**Table 6:** Factors associated with QoL in female nr-axSpA patients.

Significant Results			
Physical Component Summary			
**Associated Factors**	**β (Coefficients)**	**95% CI**	***p***
Disease Duration	0.02	0.00 to 0.03	0.049
BASDAI	−0.52	−2.78 to 1.74	0.647
ASDAS	−1.38	−4.14 to 1.38	0.320
BASFI	0.46	−0.67 to 1.59	0.423
VAS	−0.21	−1.46 to 1.04	0.739
R^2^ = 0.19; F (5,72) = 2.44; *p* = 0.046			
Mental Component Summary			
Disease Duration	0.00	−0.02 to 0.03	0.795
BASDAI	−2.30	−5.75 to 1.15	0.187
ASDAS	1.14	−3.07 to 5.35	0.590
BASFI	−0.86	−2.58 to 0.87	0.324
VAS	0.22	−1.70 to 2.13	0.821
R^2^ = 0.19; F (5,72) = 2.63; *p* = 0.043			
Non-Significant Results			
PCA	Arthritis; Entenzitis; Family History of SpA; HLA-B27; Elevated CRP; Sacroiliitis; Spondylitis		
MCS	Arthritis; Entenzitis; Family History of SpA; HLA-B27; Elevated CRP; Sacroiliitis; Spondylitis		

Abbreviations: ASDAS—Ankylosing Spondylitis Disease Activity Score; BASDAI—Bath Ankylosing Spondylitis Disease Activity Index; BASFI—Bath Ankylosing Spondylitis Functional Activity Index; β—standardized regression coefficient; CI—confidence interval; CRP—C-reactive protein; F—statistic for the overall model; HLA—Human Leukocyte Antigen; R^2^—coefficient of determination; VAS—Visual Analogue Scale; SpA—spondyloarthritis.

**Table 7:** Factors associated with anxiety and depression symptoms in female nr-axSpA patients.

Significant Results			
Hospital Anxiety and Depression Scale—Depression			
**Associated Factors**	**β (Coefficients)**	**95% CI**	***p***
Arthritis	0.19	−1.48 to 1.87	0.818
Enthesitis	1.69	−0.16 to 3.54	0.072
Family History of SpA	−1.64	−3.42 to 0.13	0.069
HLA-B27	−1.52	−3.54 to 0.5	0.137
Elevated CRP	0.23	−0.11 to 0.58	0.182
R^2^ = 0.21; F (5,72) = 2.88; *p* = 0.023			
Hospital Anxiety and Depression Scale—Depression			
Disease Duration	0.00	−0.01 to 0.01	0.801
BASDAI	1.06	−0.17 to 2.29	0.091
ASDAS	−0.16	−1.66 to 1.34	0.832
BASFI	0.33	−0.29 to 0.94	0.290
VAS	−0.37	−1.05 to 0.32	0.286
R^2^ = 0.31; F (5,72) = 4.66; *p* = 0.001			
Non-Significant results			
HADS-A	Arthritis; Entenzitis; Family History of SpA; HLA-B27; Elevated CRP; Sacroiliitis; Spondylitis; Disease duration; BASDAI; ASDAS; BASFI; VAS		
HADS-D	Sacroiliitis; Spondylitis		

Abbreviations: ASDAS—Ankylosing Spondylitis Disease Activity Score; BASDAI—Bath Ankylosing Spondylitis Disease Activity Index; BASFI—Bath Ankylosing Spondylitis Functional Activity Index; β—standardized regression coefficient; CI—confidence interval; CRP—C-reactive protein; F—statistic for the overall model; HADS-A—Hospital Anxiety and Depression Scale—Anxiety; HADS-D—Hospital Anxiety and Depression Scale—Depression; HLA—Human Leukocyte Antigen; R^2^—coefficient of determination; VAS—Visual Analogue Scale; Spa—spondyloarthritis.

## Data Availability

The data presented in this study are available on request from the corresponding author. The data are not publicly available due to privacy and ethical reasons.

## Abbreviations

The following abbreviations are used in this manuscript:

AS	Ankylosing Spondylitis
ASAS	Assessment of Spondyloarthritis International Society
ASDAS	Ankylosing Spondylitis Disease Activity Score
axSpA	Axial spondyloarthritis
BASDAI	Bath Ankylosing Spondylitis Disease Activity Index
BASFI	Bath Ankylosing Spondylitis Functional Activity Index
BASRI	Bath Ankylosing Spondylitis Radiology Index
CI	Confidence interval
CRP	C-reactive protein
DMARDs	Disease-Modifying Antirheumatic Drugs
DRVs	Disease-related variables
ESR	Erythrocyte sedimentation rate
HADS	Hospital Anxiety and Depression Scale
HADS-A	Hospital Anxiety and Depression Scale—Anxiety
HADS-D	Hospital Anxiety and Depression Scale—Depression
HLA	Human Leukocyte Antigen
IBD	Inflammatory bowel disease
IBP	Inflammatory back pain
IL-17i	Interleukin 17 inhibitor
IQR	Interquartile range
JAKi	Janus Kinase Inhibitor
MCS	Mental Component Summary
MRI	Magnetic resonance imaging
n	Number of cases
N	Total number
nr-axSpA	Non-radiographic axial spondyloarthritis
NSAIDs	Non-steroidal anti-inflammatory drugs
PCS	Physical Component Summary
QoL	Quality of life
SD	Standard deviation
SF-36	Short Form-36
SpA	Spondyloarthritis
SpA-fs	Spondyloarthritis features
TNFi	Tumor Necrosis Factor Inhibitor
VAS	Visual Analogue Scale
